# Psychological and somatic distress profiles in young adults in and out of conflict settings: a cluster-based analysis of three population-based studies in Ukraine and Switzerland

**DOI:** 10.3389/fpubh.2026.1922204

**Published:** 2026-09-24

**Authors:** Ann-Kathrin Schwientek, Julia Braun, Andreas M. Baumer, Viktoriia Yasenok, Viktoriia Petrashenko, Marco Kaufmann, Anja Frei, Seraina Rüegger, Tala Ballouz, Andrii Loboda, Vladyslav Smiianov, Susi Kriemler, Viktor von Wyl, Susanne Walitza, Andriana Kostenko, Stefan Büchi, Milo A. Puhan

**Affiliations:** 1 Epidemiology, Biostatistics and Prevention Institute, University of Zurich, Zurich, Switzerland; 2 Institute for Implementation Science in Health Care, University of Zurich, Zurich, Switzerland; 3 Population Research Center, University of Zurich, Zurich, Switzerland; 4 Sumy State University, Sumy, Ukraine; 5 Department of Child and Adolescent Psychiatry and Psychotherapy, University of Zurich, Zurich, Switzerland; 6 MediX Gruppenpraxis, Zurich, Switzerland

**Keywords:** anxiety, bodily distress disorder, cluster analysis, depression, population study, posttraumatic stress disorder, somatic symptoms, young adults

## Abstract

**Background:**

Young adulthood is a developmental period characterized by major life transitions and increased exposure to stress, during which vulnerability to psychological and somatic symptoms may be heightened. These symptoms frequently co-occur, posing challenges for diagnostic and treatment approaches. Despite the mental and physical symptom burden among young adults, population-based data on symptom patterns in this age group remains sparse. This study investigates psychological and somatic symptom patterns among young adults in three populations with differing socio-geographic and war-related context: young adults in Switzerland, Ukrainians who fled to Switzerland, and Ukrainians living in Ukraine.

**Methods:**

We analyzed cross-sectional baseline data from the Mental Health Assessment of the Population (MAP) studies, where we enrolled randomly selected young adults aged 18–24 from Switzerland, Ukrainian refugees in Switzerland, and Ukrainians residing in regions with different degrees of proximity to active war zones. Participants completed standardized self-reported screening instruments assessing depressive symptoms (PHQ-9), anxiety symptoms (GAD-7), trauma-related stress symptoms (PCL-5), and somatic symptoms (PHQ-15). Descriptive statistics were calculated, and correlations between symptom domains were examined using Spearman correlations. K-means clustering was conducted separately in each subsample to characterize patterns of psychological and somatic symptom severity, with the number of clusters selected using the gap statistic.

**Results:**

The baseline sample comprised *n* = 142 participants in MAP-Z (ZH), *n* = 102 in MAP-Z (UA), and *n* = 1,176 in the MAP-U subgroup. Complete PHQ-9, GAD-7, PCL-5, and PHQ-15 scores were available for 1,181 participants included in the cluster analyses (Switzerland: *n* = 127; Ukrainian refugees in Switzerland: *n* = 91; Ukraine: *n* = 963). Across samples, psychological symptoms showed moderate to strong correlations with somatic symptoms (Spearman's r = 0.53–0.69). For all subgroups, three clusters were identified as optimal by the gap statistic. These clusters were differentiated primarily by overall symptom burden, forming low, moderate, and high general distress profiles across psychological and somatic symptom domains. Similar severity-graded patterns were observed in all three samples.

**Conclusion:**

Across all three samples, psychological and somatic symptoms co-occurred and were differentiated by overall symptom severity. These findings suggest that assessments in young adults should consider symptom burden across multiple domains. Severity-based symptom patterns may also help inform targeted prevention and early-intervention strategies.

## Introduction

Psychological symptoms associated with depression, anxiety, and trauma-related stress frequently co-occur with persistent somatic symptoms (1–3), posing complex diagnostic and therapeutic challenges for health care professionals, particularly in primary care settings (4–6). Young adulthood is a developmental stage characterized by major life transitions, physiological and psychosocial changes, and increased stress exposure, where vulnerability to psychological and somatic symptoms is heightened and may carry long-term implications for health and functioning (3, 7–9). International data indicate a high burden of mental health problems and physical symptoms among adolescents and young adults (3, 7, 10–14). Even mild complaints can impair daily functioning and are associated with poorer health status and increased healthcare utilization, regardless of underlying medical pathology (6, 13, 15). Moreover, young adults with mental health problems and chronic conditions may face substantial difficulties entering the labor market and remaining in employment, with far-reaching economic consequences (7, 16, 17). In Switzerland, despite an overall decline in disability pension inflows, disability pension rates among young adults have not decreased (17).

The overlap between somatic symptoms and mental health conditions further complicates differential diagnosis and treatment planning (18–20).

For many common mental health conditions, evidence suggests that psychopathological symptoms may be better characterized along continuous dimensions than by strictly discrete diagnostic boundaries, questioning the usefulness of diagnostic categories for treatment-selection and prognosis (19, 21–25). These findings have contributed to ongoing debate about the limitations of categorical approaches, including high levels of comorbidity, within-diagnosis heterogeneity, and potentially limited correspondence with prognosis and treatment response (22, 23, 26). Despite longstanding criticism of strictly categorical approaches to psychopathology, formal diagnostic classification systems such as the International Classification of Diseases (ICD) and the Diagnostic and Statistical Manual of Mental Disorders (DSM) continue to play a central role in routine psychiatric practice, including diagnosis, clinical communication, and administrative coding (22, 27).

Recent revisions have begun to incorporate dimensional elements; ICD-11, for example, includes dimensional approaches for selected disorder groups, while both DSM-5 and ICD-11 have revised the conceptualization of somatic symptom-related conditions, moving away from the requirement that symptoms be medically unexplained (28–30). Transdiagnostic approaches, such as the Hierarchical Taxonomy of Psychopathology (HiTOP) model, the Research Domain Criteria (RDoC) matrix, and the Network Theory (NT), emphasize shared underlying mechanisms and better capture the complexity and comorbidity observed in clinical practice (31–38).

Despite increasing evidence of overlap across symptom domains, comparatively little is known about how psychological and somatic symptom burden jointly manifests among young adults at the population level, particularly across populations exposed to different social and environmental contexts. Evidence further suggests that psychological distress in youth is multidimensional and heterogeneous (39). This gap is notable given evidence of a high somatic symptom burden among youth with mental disorders (11, 13, 40, 41). A better understanding of the co-occurrence and distribution between psychological and somatic symptoms may provide a more comprehensive characterization of symptom burden in young adults and inform early detection, diagnostic approaches, and integrated care strategies (6, 10, 42).

The present study aimed to characterize the co-occurrence of self-reported symptoms related to depression, anxiety, trauma-related stress, as well as somatic symptoms, and to identify patterns of symptom burden among young adults. It further sought to extend previous work by examining populations with different environmental contexts, including war- and non-war-related settings.

## Material and methods

### Study design and setting

We used baseline data from the MAP studies (43), which are prospective, population-based, observational cohort studies encompassing three populations: the general population of Zurich, Switzerland [MAP-Z (ZH)], refugees from Ukraine residing in the Canton of Zurich [MAP-Z (UA)], and the general population of Ukraine (MAP-U). With the support of nearly three dozen stakeholders from Switzerland and Ukraine, including representatives from more than five Swiss universities and organizations, Ukrainian universities, the International Red Cross, the Psychiatric Hospital of the University of Zurich, the University Children's Hospital Zurich, and public health authorities from both countries, we held a full-day working session in March 2023 to finalize a mental health digital surveillance protocol (MAP studies) tailored to the Ukrainian context. The participating experts included specialists in public health, mental health, political science, and health policy, as well as representatives of Ukrainian and Swiss governmental and non-governmental organizations, including the International Red Cross and UNESCO, social media specialists, and Ukrainian citizens (43). For additional detailed information about the study design and the rationale of the sample size calculation, we refer to the MAP protocol and baseline paper (43, 44).

In brief, the MAP studies aim to enable public mental health surveillance through online monitoring of psychological symptoms among the general population and relevant subgroups.

To better reflect the different regional levels of war-related exposure within Ukraine (MAP-U), we focused on three regional clusters across different parts of the country, each experiencing varying degrees of impact from the ongoing war. At the time of recruitment, the North-West cluster (NW), including Rivne, Lviv, and Chernivtsi oblasts, was located far from the active war zone and experienced comparatively low security risks.

The Central cluster (CE), including Zhytomyr, Kyiv, and Dnipropetrovsk oblasts, was closer to the border with Russia with moderate security risks. The South-East cluster (SE), including Sumy, Kharkiv, Mykolaiv, and Kherson oblasts, was situated near active war zones, facing high security risks (Supplementary Figure S1). The above described geographical clustering of Ukrainian regions was developed in collaboration with researchers from Sumy State University during the study-design phase in 2023–2024. It reflected the regional and security context at that time as a grouping for geographically related war experiences and was applied to data collected during the 2024 baseline assessment. Further details on the rationale underlying the regional grouping are provided in the MAP protocol and baseline paper (43, 44). The analysis in this paper focuses on the subgroup of young adults aged 18–24 years. Accordingly, we describe only the methods and results relevant to this study population.

### Eligibility criteria and recruitment

Participants were recruited using population-based sampling frameworks, with eligible individuals selected from defined source populations. The study enrolled participants aged ≥ 18 years, capable of completing an online survey and providing informed consent. For MAP-Z (ZH) we included individuals residing in the Canton of Zurich, holding either Swiss citizenship or residence permit B (time-limited residence and work permit for foreign nationals staying in Switzerland for more than a year) or C (settlement permit that grants permanent residency rights). Participants in MAP-Z (UA) were Ukrainian nationals in the canton of Zurich, registered with protection status S (temporary residency permit granted to individuals fleeing conflict until returning home is considered safe). For MAP-U, eligible participants were living in one of the 10 oblasts of Ukraine described above. Repeated unsuccessful contact attempts lead to an exclusion of the study. Recruitment was conducted between March 25 2024 and August 9 2024.

The study followed established digital cohort approaches, enabling geographically independent recruitment and a broader catchment area (45, 46). We employed age-stratified random sampling based on population registries and made substantial efforts to enhance participation rates. Potential MAP-Z (ZH) and MAP-Z (UA) participants were randomly selected by the Swiss Federal Statistical Office, and initial invitations were sent by postal mail. Potential MAP-U participants were randomly selected by the Center for Social Research at Sumy State University based on population registries. Selected persons were contacted via email, including two reminders. If there was no response, interviewers from local teams in Ukraine approached them via Telegram (until May 2024 when Ukrainians were recommended to stop using it), Viber (from May 2024) or phone calls. All recruited participants received information about the study, an invitation to participate, and a personalized link and access code to a secure digital study platform (REDCap) (47, 48). Those interested provided informed consent to participate in the study electronically via REDCap and were forwarded to the baseline assessment. Eligible for inclusion in the present analysis were all participants who had completed the baseline assessment.

### Data collection and instruments

We collected sociodemographic data and assessed health outcomes, including symptoms related to PTSD (Posttraumatic Stress Disorder Checklist, PCL-5), anxiety (Generalized Anxiety Disorder 7-item scale, GAD-7), depression (Patient Health Questionnaire-9, PHQ-9), and somatic distress (Patient Health Questionnaire-15, PHQ-15).

As clinical diagnoses could not be established by mental health professionals using ICD-10/11 (49, 50) or DSM-5 criteria (51) within the study design, mental health symptoms were assessed using standardized, widely used screening questionnaires. Therefore, results of this assessment describe symptom severity rather than formal diagnosis (52). The instruments were selected based on a selection process, detailed in our MAP study protocol (43), using psychometric quality, established instrument cut-offs, digital feasibility, population relevance and prior use in Ukraine. The selection of mental health domains was additionally informed by established international disease classifications and Global Burden of Disease (GBD) frameworks, with the aim of ensuring comparability with commonly assessed mental health conditions in population health research (53).

The PCL-5 screening instrument was administered without a separate Criterion A assessment and without anchoring responses to a standardized index event (54, 55). War-related experiences were assessed separately in the Ukrainian and refugee samples but were not linked to the PCL-5 responses. This decision was made based on ethical and methodological considerations. First, given data collection among participants living in a war-affected setting, detailed assessment of potentially traumatic life events was omitted to minimize unnecessary distress or triggering during a self-administered survey. Furthermore, omission of the additional Criterion A assessment reduced respondent burden answering a survey containing multiple instruments, which was considered important in the longitudinal MAP studies to limit fatigue and facilitate continued participation. The PCL-5 was therefore used only to quantify self-reported PTSD-related symptom severity rather than establish individual clinical diagnoses.

The Ukrainian versions of the PCL-5, PHQ-9, and GAD-7 were based on previously translated and culturally adapted versions described by Roberts et al. (2019) (56). The Ukrainian PCL-5 has demonstrated good reliability and construct validity in a Ukrainian sample and has been used in the Ukrainian context (56, 57). Ukrainian versions of the PHQ-9 and GAD-7 have also been used in previous studies involving Ukrainian populations (56, 58, 59). However, to our knowledge, formal validation of these versions in the specific population investigated in the present study has not been reported. The Ukrainian version of the PHQ-15 was developed through a process of duplicate independent translations followed by creating consensus-derived, culturally appropriate final versions. The original English-language PHQ-15 has demonstrated satisfactory validity for assessing the severity of somatic symptoms (60), however, formal validation evidence for the Ukrainian version in the present target population remains limited (43).

We also assessed the preexisting chronic physical and mental health conditions of the participants as part of the baseline assessment.

Questionnaire total scores were calculated when at least 80% of the instrument's items had been completed. When fewer than 20% of the items were missing, the missing responses were replaced with the participant's mean response across the completed items of the same instrument before calculating the total score. When 20% or more of an instrument's items were missing, no total score was calculated for that instrument. Only participants with complete sets of all four self-reported health outcome questionnaires (PHQ-9, PHQ-15, GAD-7 and PCL-5) were included in our analyses.

In our study population, internal consistency reliability was high: PHQ-9 (α = 0.86), GAD-7 (α = 0.90), PCL-5 (α = 0.94), and PHQ-15 (α=0.82). Detailed information on the cut-points for each instrument, their sensitivity and specificity, is provided in the Supplementary material.

### Statistical analysis

Analyses were conducted without sampling or nonresponse weights because the primary objective was to examine symptom patterns and associations within the study samples rather than to estimate population prevalence. Accordingly, all findings are interpreted as sample-based estimates and not as population-representative estimates.

Descriptive analyses were conducted using means with standard deviations (SD), medians and interquartile ranges (IQR) for continuous data and sum scores, and absolute numbers with percentages for categorical data. Correlations between sum scores of PHQ-15, PHQ-9, GAD-7 and PCL-5 were calculated using Spearman's correlation coefficient.

Between-group comparisons across our three samples are presented descriptively, and no inferential statistical tests or adjusted comparative analyses were performed. Given differences in demographics, recruitment, and socioeconomic characteristics between the study groups, the observed variations should be interpreted as descriptive characteristics of the respective samples and not as statistically established or causal group differences.

To identify symptom burden patterns, we performed k-means clustering using all individual items of the PHQ-15, PHQ-9, GAD-7 and PCL-5 in the MAP-Z (ZH) sample. Note that although these items are measured on an ordinal scale, we treat them as quasi-continuous. Prior to the cluster analysis, the items were standardized to z-scores. K-means clustering was performed using the Euclidean distance, with cluster centroids initialized from k = 3 randomly selected observations. The procedure was repeated 100 times with different random starts, and the solution with the lowest within-cluster sum of squares was retained. Convergence was assessed according to the Hartigan–Wong algorithm.

The gap statistic was used to determine the number of clusters, which compares the observed within-cluster dispersion with that expected under a reference distribution without cluster structure (61). Based on this criterion, we selected k=3 for all groups. Its simulation-based uncertainty reflects the robustness of the selected cluster number but not the stability of individual cluster assignments. The gap statistic identifies the point at which adding further clusters no longer improves within-cluster compactness substantially beyond what would also occur in randomly generated data. This method can be applied with different clustering algorithms, including k-means clustering.

Cluster validity was assessed by looking at the within cluster sum of squares as a measure of within-cluster dispersion as well as the between-cluster sum of squares and the percentage of the variation explained by the clustering. In addition, we looked at mean silhouette coefficients to evaluate cluster separation, where a value of 1 stands for perfect classification, a value of 0 for random classification and a value of−1 represents bad classification, where an individual should actually be found in a different cluster. Finally, we report mean Jaccard coefficients calculated using 500 bootstrap samples to assess cluster stability. These coefficients can take values between 0 and 1, where a value below 0.6 represents instability.

We applied the same cluster analysis approach and steps to Ukrainian participants, both residing in Switzerland [MAP-Z (UA)] and living in different regions of Ukraine (MAP-U). This enabled us to investigate symptom severity patterns across different populations.

For MAP-U sample we performed all data analyses stratified by the three Ukrainian regions (North-West, Central, South-East) describing the results across the different subsamples for different geographical war-related exposure.

We conducted a full-case analysis. In order to assess the outer bounds of the impact of the exclusions of missing and partially missing answers, we conducted two extreme value sensitivity analyses. We replaced all missing responses with 0 and with the maximum scale value and compared the identified clusters qualitatively with our full-scale analysis. Additionally, we looked at hierarchical clustering as an alternative clustering approach and compared the resulting clusters using the adjusted Rand index and the percentage of equal classifications.

Direct item overlap was defined conservatively as the inclusion of the exact same symptom item in more than one scale score. Although some items assessed highly related constructs, particularly irritability and heightened arousal, they differed in wording, the kind of information that was assessed, reference period, and clinical context and were therefore classified as conceptually rather than directly overlapping. As a sensitivity analysis, we examined whether direct item overlap between the PHQ-9 and PHQ-15 (the sleep and fatigue items) as well as PHQ-9 and PCL-5 (concentration) contributed to the identification of clusters. Accordingly, the PHQ-9 components assessing sleep disturbance, fatigue or low energy, and concentration difficulties were excluded from the PHQ-9 score. Conceptually related but non-identical items across the GAD-7, PCL-5, PHQ-9, and PHQ-15 were retained. The resulting set of overlap-reduced PHQ-9 items was standardized and entered together with the standardized PHQ-15, GAD-7, and PCL-5 items into the same k-means clustering procedure used in the primary analysis.

We conducted all analyses in R 4.5.2.

## Results

### Sample characteristics and descriptive analysis

Information about the recruitment process, recruitment challenges, and participation rates can be found in our baseline as well as recruitment challenge paper (44, 62). Participation differed across study populations. For MAP-U, an exact response rate could not be determined because the number of sampled individuals who were successfully reached during the ongoing war could not be established. Participants were recruited from all predefined geographical regions of Ukraine. Response rates in the two Swiss samples were low to moderate.

The baseline sample comprised a total of *n* = 142 participants in MAP-Z (ZH), *n* = 102 in MAP-Z (UA), and *n* = 1,176 in the MAP-U subgroup. Complete PHQ-9, GAD-7, PCL-5, and PHQ-15 scores were only available for 1,181 participants, including MAP-Z (ZH): *n* = 127; MAP-Z (UA): *n* = 91 and MAP-U: *n* = 963. We descriptively analyzed data from all participants who have fully completed the self-reported questionnaires (full-case analysis) as this is required for the cluster analysis. Sociodemographic characteristics and existing chronic health conditions of young adults among the different subgroups are detailed in Table 1. The same information for the excluded participants can be found in Supplementary Table S1, and the number of missing values per instrument in the excluded participants in Supplementary Table S2.

**Table 1:** Demographic characteristics and health conditions of MAP-Z (ZH), MAP-Z (UA), MAP-U age grouped 18–24 years old (full-case analysis, in total *n* = 1,181 participants).

Characteristics	MAP-Z (ZH) (*n* = 127)	MAP-Z (UA) (*n* = 91)	MAP-U (*n* = 963)		
			North-West (*n* = 211)	Central (*n* = 230)	South-East (*n* = 522)
Sex					
Female	73 (57.5%)	66 (72.5%)	154 (73.0%)	178 (77.4%)	356 (68.2%)
Male	52 (40.9%)	25 (27.5%)	57 (27.0%)	52 (22.6%)	164 (31.4%)
Other	2 (1.6%)	0 (0.0%)	0 (0.0%)	0 (0.0%)	2 (0.4%)
Highest educational attainment					
Less than secondary education	1 (0.8%)	0 (0.0%)	1 (0.5%)	1 (0.4%)	0 (0.0%)
Completed secondary education	23 (18.1%)	32 (35.2%)	53 (25.1%)	104 (45.2%)	198 (37.9%)
Professional education	32 (25.2%)	13 (14.3%)	38 (18.0%)	40 (17.4%)	64 (12.3%)
Baccalaureate or higher professional education^1^	48 (37.8%)	NA	NA	NA	NA
University degree	23 (18.1%)	45 (49.5%)	119 (56.4%)	84 (36.5%)	259 (49.6%)
Missing	0 (0%)	1 (1.1%)	0 (0%)	1 (0.4%)	1 (0.2%)
Existing chronic physical conditions					
Yes^2^	9 (7.1%)	22 (24.2%)	53 (25.1%)	76 (33.0%)	177 (33.9%)
No	117 (92.1%)	69 (75.8%)	155 (73.5%)	148 (64.3%)	339 (64.9%)
Missing	1 (0.8%)	1 (1.1%)	3 (1.4%)	6 (2.6%)	6 (1.1%)
Existing chronic mental health conditions					
Yes	19 (15.0%)	11 (12.1%)	10 (4.7%)	17 (7.4%)	35 (6.7%)
No	107 (84.3%)	79 (86.8%)	198 (93.8%)	207 (90.0%)	571 (92.1%)
Missing	1 (0.8%)	1 (1.0%)	3 (1.5%)	6 (2.6%)	6 (1.1%)

^1^Because education systems differ greatly, no direct equivalent exists in all cases. ^2^Chronic physical health conditions include cardiovascular, neurological, stroke, respiratory, oncological, diabetes, digestive, genitourinary, musculoskeletal, allergic conditions. NA, Not Applicable; MAP-Z (ZH), Mental Health Assessment of the Swiss Population in Zurich; MAP-Z (UA), Mental Health Assessment of the Ukrainian Population in Zurich; MAP-U, Mental Health Assessment of the Population in Ukraine.

In the data we analyzed, a sex imbalance with more women (57.5% to 77.4%) was observed in all samples. Across all study arms between 22.0% to 40.6% of the young adults reported preexisting chronic health conditions, including physical (cardiovascular, neurological, stroke, respiratory, oncological, diabetes, digestive, genitourinary, musculoskeletal, allergic conditions) and mental health conditions.

Supplementary Table S3 in the supplementary material summarizes direct and indirect (family-related) war exposure in the MAP-Z (UA) and MAP-U samples, providing background information for these study groups.

Cross tabulation of moderate to severe somatic symptoms and elevated psychological symptoms using established screening tool cut-offs are presented also in the supplementary material (Supplementary Tables S4–Table S8). The results indicate moderate to high co-occurrence of moderate to severe somatic, depression-related, anxiety-related, and PTSD-related symptoms among all study arms [MAP-Z (ZH), MAP-Z (UA), MAP-U (NW), MAP-U (CE) and MAP-U (SE)].

An overview of symptom burden across all subgroups of young adults is detailed in Figure 1 for somatic, depression-related, anxiety-related and PTSD-related symptoms. Across all subgroups boxplots showed a low to moderate symptom burden. Detailed results for all sum scores across instruments and subgroups can be found in the supplementary material (Supplementary Table S9). Age- and sex-stratified patterns of symptom burden across the subpopulations were visually examined using boxplots and have been reported in our baseline publication (44).

**Figure 1:**
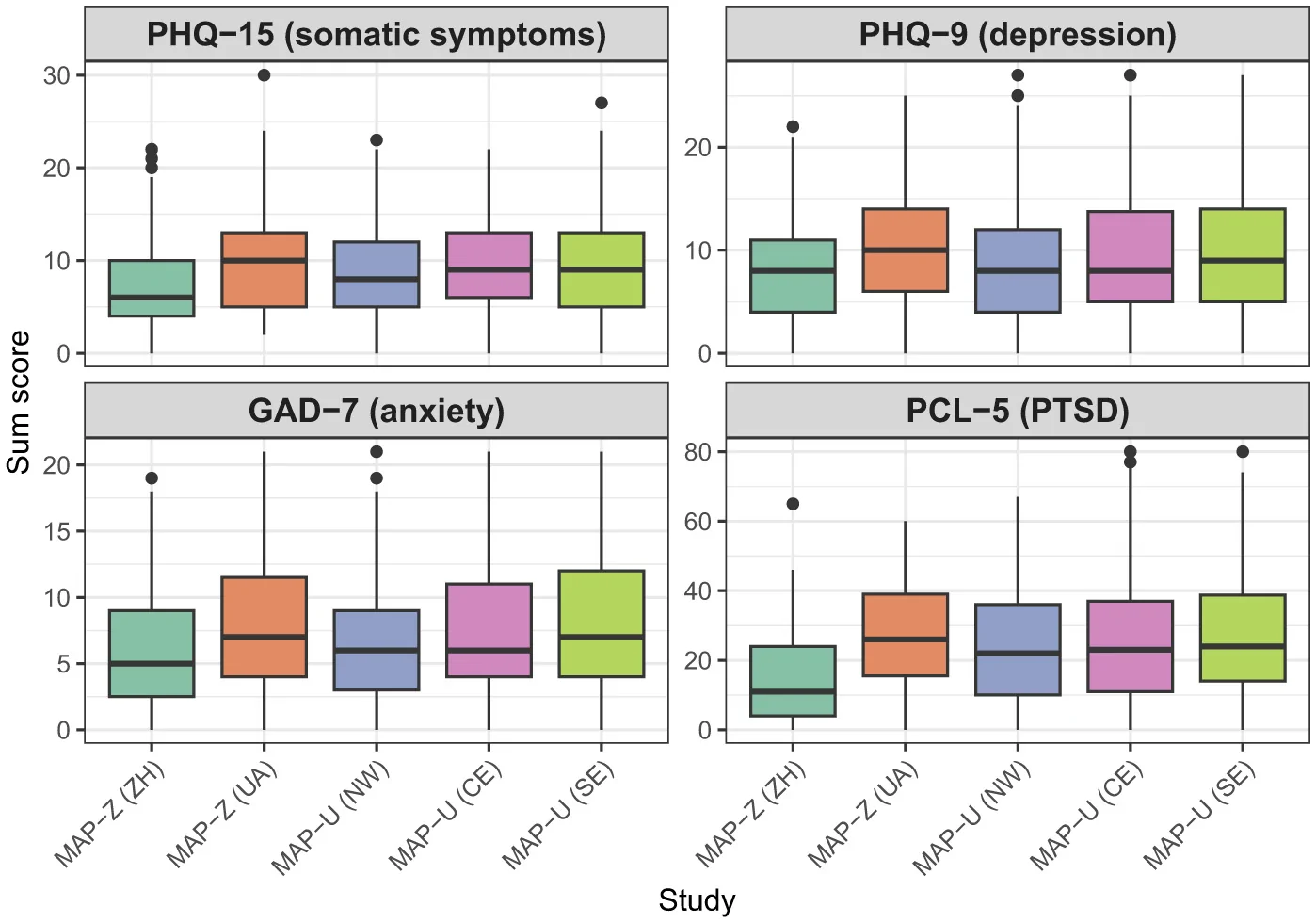
Boxplots of PHQ-15, PHQ-9, GAD-7 and PCL-5 sum scores by MAP study arms MAP-U (NW), MAP-U (SE), MAP-U (CE), MAP-Z (UA) and MAP-Z (ZH) 18–24 years old. MAP-Z (ZH), Mental Health Assessment of the Swiss Population in Zurich; MAP-Z (UA), Mental Health Assessment of the Ukrainian Population in Zurich; MAP-U (NW), Mental Health Assessment of the Population in Ukraine (North-West); MAP-U (CE), Mental Health Assessment of the Population in Ukraine (Central); MAP-U (SE), Mental Health Assessment of the Population in Ukraine (South-East); PHQ-15, Patient Health Questionnaire-15; PHQ-9, Patient Health Questionnaire-9; GAD-7, Generalized Anxiety Disorder-7; PCL-5, PTSD Checklist for DSM-5.

In the supplementary material, we provide an overview of the frequency of individual items from the PHQ-15 reported in the different subgroups (Supplementary Figures S2–S4). Among all study arms the most frequently reported somatic complaints were fatigue, sleep difficulties, back pain and headaches.

### Main results

The correlations between somatic and psychological symptoms are illustrated in scatterplots in Figure 2. PHQ-15 sum scores were positively correlated with depressive (PHQ-9), anxiety (GAD-7), and PTSD (PCL-5) symptom scores. Correlations across all instruments and subgroups ranged from r = 0.53 to r = 0.69 (moderate to strong correlations).

**Figure 2:**
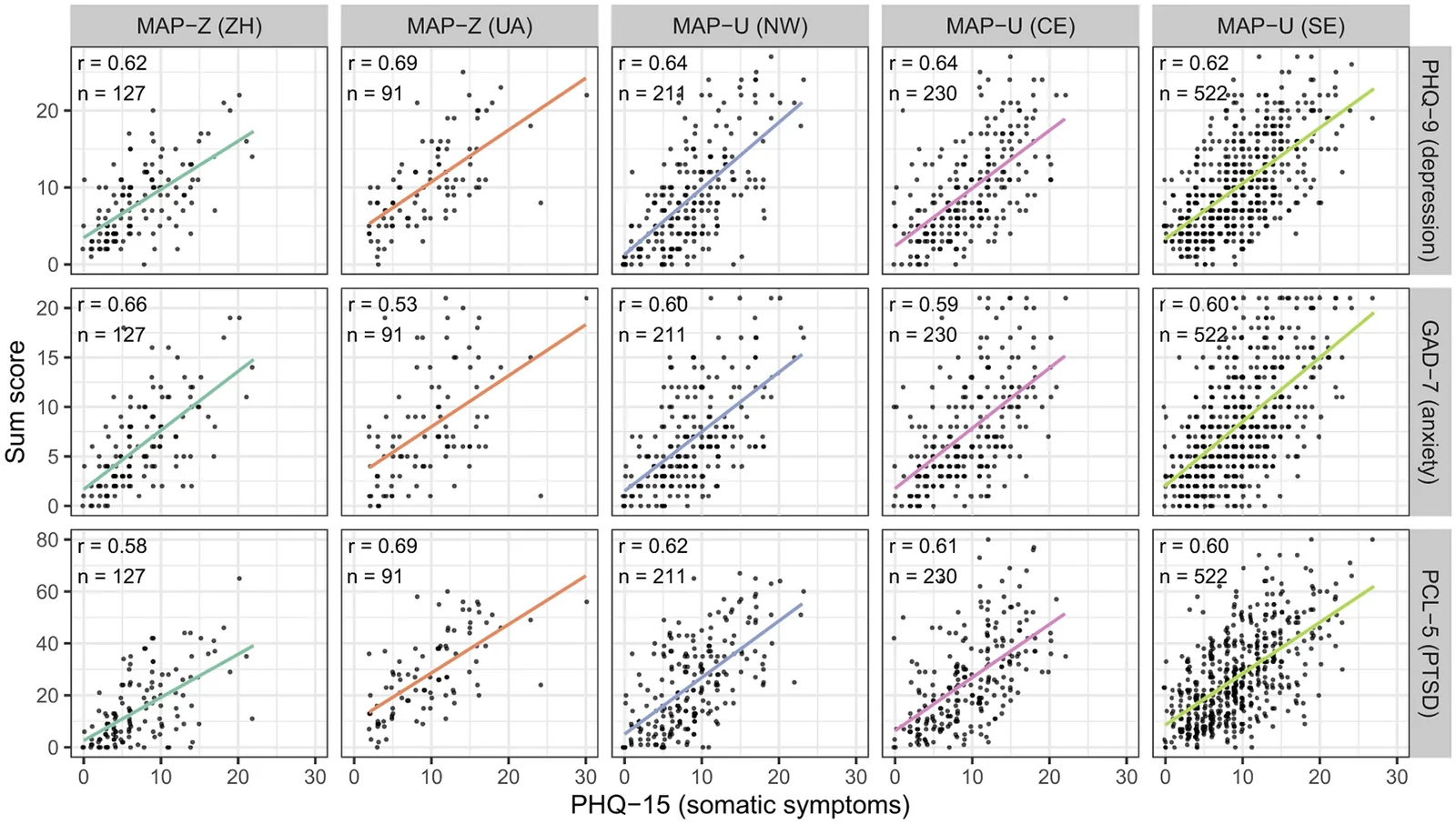
Scatterplots with regression lines describing the correlation between PHQ-15 with PHQ-9, GAD-7 and PCL-5 sum scores in MAP-Z (ZH), MAP-Z (UA), MAP-U (NW), MAP-U (CE) and MAP-U (SE). MAP-Z (ZH), Mental Health Assessment of the Swiss Population in Zurich; MAP-Z (UA), Mental Health Assessment of the Ukrainian Population in Zurich; MAP-U (NW), Mental Health Assessment of the Population in Ukraine (North-West); MAP-U (CE), Mental Health Assessment of the Population in Ukraine (Central); MAP-U (SE), Mental Health Assessment of the Population in Ukraine (South-East); PHQ-15, Patient Health Questionnaire-15; PHQ-9, Patient Health Questionnaire-9; GAD-7, Generalized Anxiety Disorder-7; PCL-5, PTSD Checklist for DSM-5.

To further identify symptom patterns for all five subgroups, three clusters were identified as optimal by the gap statistic. The cluster analysis required valid PHQ-9, PHQ-15, GAD-7, and PCL-5 sum scores. Consequently, 1,181 participants were included: 127 of 142 participants in MAP-Z (ZH) (89%), 91 of 102 in MAP-Z (UA) (89%), and 963 of 1,176 in MAP-U (82%). Overall, 239 participants, corresponding to 16.8% of the baseline sample, were excluded.

This led to the following number of participants in each cluster:

MAP-ZH: total: *n* = 127; Cluster 1: *n* = 34 (27%), Cluster 2: *n* = 42 (33%), Cluster 3: *n* = 51 (40%)
MAP-ZH (UA): total: *n* = 91; Cluster 1: *n* = 16 (18%), Cluster 2: *n* = 33 (36%), Cluster 3: *n* = 42 (46%)
MAP-U (NW): total: *n* = 211; Cluster 1: *n* = 31 (15%), Cluster 2: *n* = 83 (39%), Cluster 3: *n* = 97 (46%)
MAP-U (CE): total: *n* = 230; Cluster 1: *n* = 61 (27%), Cluster 2: *n* = 86 (37%), Cluster 3: *n* = 83 (36%)
MAP-U (SE): total: *n* = 522; Cluster 1: *n* = 106 (20%), Cluster 2: *n* = 207 (40%), Cluster 3: *n* = 209 (40%)

Clusters demonstrated three general distress levels across domains, as Cluster 1 (high-distress group), Cluster 2 (moderate-distress group) and Cluster 3 (low-distress group) (Figure 3). Cluster 1 (high-distress group) showed elevated scores across nearly all symptom domains, particularly for anxiety-related variables (e.g., afraid, worried), depressive symptoms (e.g., failure, tired), and PTSD stress reactivity (e.g., feel stress again, trouble falling asleep) and somatic complaints such as headache and fatigue. Most items were markedly above the average behavior of the participants, with values clearly above 0.5 SD and partly reaching a value up to one SD. This pattern suggests a generalized distress cluster characterized by high comorbidity and symptom overlap across various symptom domains. Cluster 2 exhibited a more moderate symptom structure, with most symptom scores falling between the population mean and 0.5 SD above it, thus not being markedly different from the average values of the participants. This group can be characterized as a moderate-distress group. Cluster 3 showed a low symptom burden across all domains, with all symptoms scoring consistently below the mean, however, not as strongly on the negative side as the high distress group is on the other side, with values up to 0.5 SD lower than the mean. This group can be therefore interpreted as a low-symptom cluster, reflecting low anxiety-related, depression-related, somatic, and PTSD-related symptomatology.

**Figure 3:**
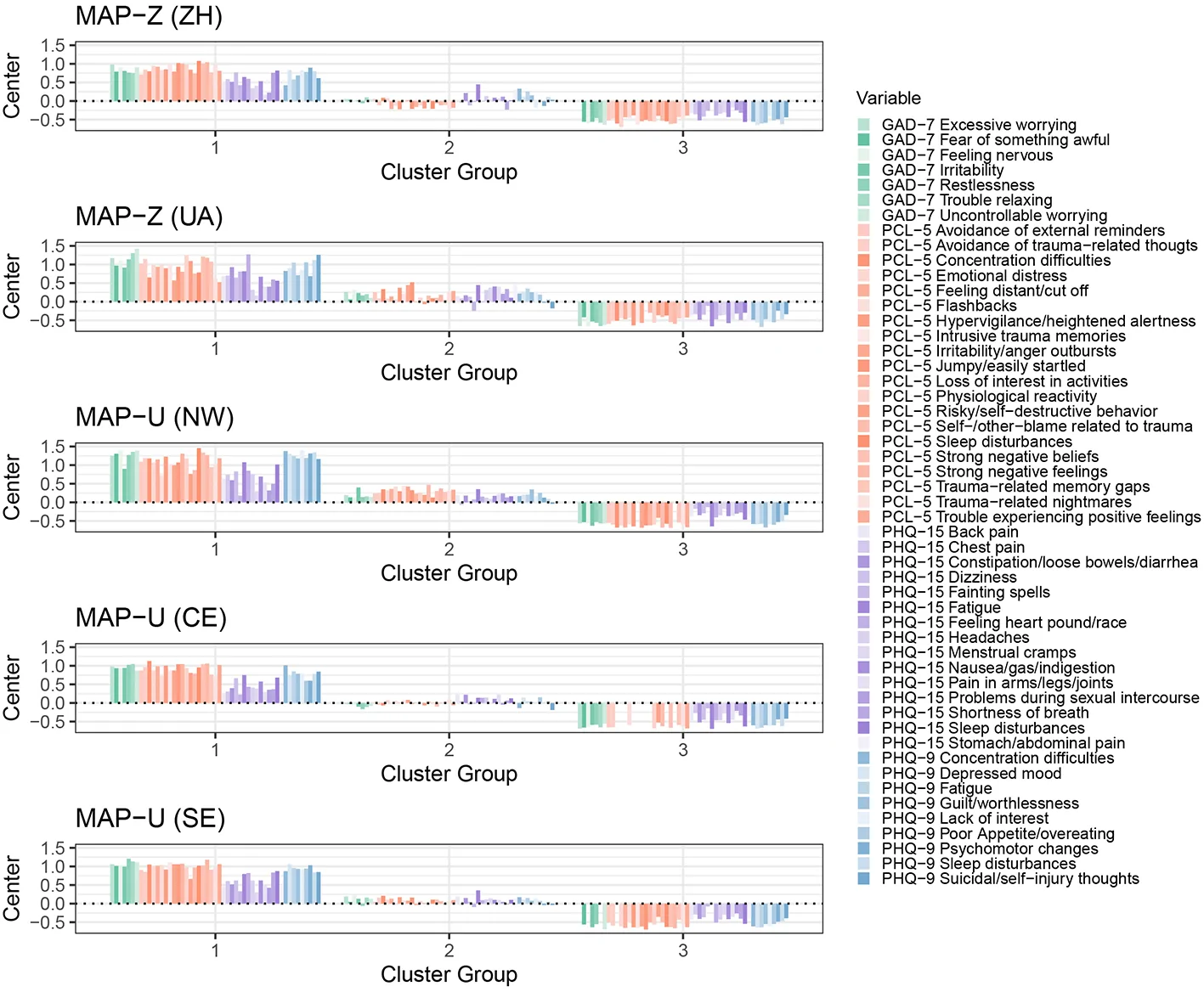
K-means cluster analysis (3 clusters) including PHQ-15, PHQ-9, GAD-7 and PCL-5 items for MAP-Z (ZH), MAP-Z (UA), MAP-U (SE), MAP-U (CE) and MAP-U (NW). The figure shows for each item the standardized distance from the center of each cluster. This means that positive values occurred more strongly than average, whereas negative values represented lower values compared to the mean behavior. MAP-Z (ZH), Mental Health Assessment of the Swiss Population in Zurich; MAP-Z (UA), Mental Health Assessment of the Ukrainian Population in Zurich; MAP-U (NW), Mental Health Assessment of the Population in Ukraine (North-West); MAP-U (CE), Mental Health Assessment of the Population in Ukraine (Central); MAP-U (SE), Mental Health Assessment of the Population in Ukraine (South-East); PHQ-15, Patient Health Questionnaire-15; PHQ-9, Patient Health Questionnaire-9; GAD-7, Generalized Anxiety Disorder-7; PCL-5, PTSD Checklist for DSM-5.

We applied the same cluster analysis approach to Ukrainian participants, both residing in Switzerland and in different regions of Ukraine (also shown in Figure 3). Across all subsamples, a 3-cluster solution provided the best fit, consistently identifying a high-distress group (Cluster 1), a moderate-distress group (Cluster 2), and a low-distress group (Cluster 3). The sizes of the resulting clusters are given in Table 2.

**Table 2:** Average symptom sum scores, standard deviation and cluster size for the three clusters across subgroups [MAP-Z (ZH), MAP-Z (UA), MAP-U (NW), MAP-U (CE) and MAP-U (SE)].

Cluster	Instrument	MAP-Z (ZH)	MAP-Z (UA)	MAP-U (NW)	MAP-U (CE)	MAP-U (SE)
1	PHQ-15	12.1 (4.7)	15.9 (5.1)	15.0 (4.3)	13.9 (4.1)	15.0 (4.5)
1	PHQ-9	13.4 (4.2)	18.8 (4.1)	20.1 (4.1)	16.4 (4.8)	18.2 (4.1)
1	GAD-7	11.2 (3.6)	15.9 (3.2)	14.8 (4.1)	14.0 (4.2)	15.5 (3.9)
1	PCL-5	33.2 (9.7)	51.6 (5.7)	50.7 (10.3)	49.0 (11.7)	51.8 (10.4)
2	PHQ-15	8.2 (3.4)	12.2 (3.8)	10.2 (3.8)	10.6 (3.8)	10.4 (3.7)
2	PHQ-9	8.8 (2.7)	12.0 (3.0)	10.4 (3.5)	9.7 (4.0)	10.8 (3.8)
2	GAD-7	6.3 (3.0)	9.2 (4.4)	8.0 (3.1)	7.1 (3.0)	8.9 (3.5)
2	PCL-5	13.0 (7.1)	32.9 (8.8)	31.7 (8.5)	26.1 (7.1)	29.0 (8.1)
3	PHQ-15	3.7 (2.3)	5.5 (2.9)	5.5 (3.2)	5.5 (3.1)	5.3 (3.0)
3	PHQ-9	4.1 (2.3)	6.3 (3.0)	3.8 (3.2)	4.4 (2.6)	5.2 (2.9)
3	GAD-7	2.6 (1.8)	3.8 (2.7)	3.1 (2.2)	3.4 (2.5)	3.5 (2.4)
3	PCL-5	4.4 (4.1)	15.5 (8.2)	9.1 (6.7)	9.1 (5.7)	12.4 (7.1)

MAP-Z (ZH), Mental Health Assessment of the Swiss Population in Zurich; MAP-Z (UA), Mental Health Assessment of the Ukrainian Population in Zurich; MAP-U (NW), Mental Health Assessment of the Population in Ukraine (North-West); MAP-U (CE), Mental Health Assessment of the Population in Ukraine (Central); MAP-U (SE), Mental Health Assessment of the Population in Ukraine (South-East); PHQ-15, Patient Health Questionnaire-15; PHQ-9, Patient Health Questionnaire-9; GAD-7, Generalized Anxiety Disorder-7; PCL-5, PTSD Checklist for DSM-5.

Table 2 shows the average symptom sum scores for the three clusters per subgroup. For Cluster 1, the average sum scores were high, above or close to the established screening cut-offs. For Cluster 2, the average sum scores were lower than those of Cluster 1 and higher than Cluster 3 showing moderate symptom severities. In comparison, Cluster 3 showed the lowest values with sum scores indicating mild symptom severity across all instruments.

The resulting clusters from all five subgroups showed a percentage of explained variance between 28% and 33%, representing a moderate amount of explained variance. The mean silhouette coefficients were between 0.10 and 0.18 which stands for relatively weak cluster separation and a substantial amount of overlap between the clusters. Mean Jaccard similarity coefficients had values between 0.65 and 0.97. This represents mostly high to very high cluster stability, with only two values below 0.7 which stands for moderate stability.

Replacing missing values with the highest or, respectively, lowest possible value and comparing the results qualitatively showed similar patterns. Using hierarchical clustering as an alternative clustering algorithm resulted in moderate to good comparability with an adjusted Rand index above 0.5 and a percentage of equally classified persons above 80% in the MAP-ZH, MAP-U (NW) and MAP-U (SE) cohorts. The remaining two cohorts showed adjusted Rand indices of 0.30 and 0.38 with 53% and 64% of persons classified in the same cluster. In both cohorts, the high-distress cluster remained quite similar, whereas there was overlap between the other two clusters.

Finally, the sensitivity analysis where three items from PHQ-9 having the exact same wording were removed showed adjusted Rand indices of 0.90, 0.84, 0.91, 0.93 and 0.92, standing for very good comparability.

## Discussion

This study found moderate to strong correlations between psychological and somatic symptoms in young adults, while separate cluster analyses across the examined samples identified severity-graded symptom patterns. Participants were differentiated primarily according to overall symptom burden, with low-, moderate-, and high-burden clusters. These patterns were observed across geographically and contextually distinct samples with different war-related exposures and security contexts. Three symptom severity-based clusters emerged: high, moderate, and low distress clusters. The findings of severity-based clusters should be interpreted in light of both the structure of the symptom measures and the analytical approach.

Direct and conceptual overlap between the screening instruments may have contributed to the observed findings. Such overlap is not unexpected, as depression-, anxiety-, trauma-related, and somatic symptom constructs share several clinical features and may capture partly overlapping dimensions of distress (19, 26). We distinguished between direct overlap, identified for sleep disturbance and fatigue (PHQ-9 and PHQ-15) and for concentration difficulties (PHQ-9 and PCL-5) and conceptual overlap. Other symptoms assessed across instruments, including irritability and heightened arousal, were considered conceptually overlapping rather than directly overlapping because they differed in wording, the type of information assessed, reference period, or clinical context. However, the sensitivity analysis excluding the three directly overlapping PHQ-9 items showed adjusted Rand indices of 0.84–0.93 across the five subgroups, indicating strong agreement with the primary cluster assignments. This suggests that direct item overlap alone is unlikely to account for the observed severity-based clustering pattern. Nevertheless, broader conceptual overlap between symptom constructs may still have contributed to the shared variance across psychological and somatic symptom domains.

The overlap in several of the items can also be seen in the moderate percentage of explained variance and especially in the relatively weak silhouette coefficients of the k-means cluster analysis. However, the mostly high to very high cluster stability illustrates the value of the analysis, and given the good interpretability of the resulting cluster structure, the analysis adds considerable value.

The use of k-means clustering on numerous positively correlated symptom items may have predisposed the analysis to identify groups primarily according to overall response magnitude, resulting in low-, moderate-, and high-burden clusters. The identified clusters may therefore predominantly reflect a general distress or symptom-severity dimension and do not establish the absence of disorder-specific symptom profiles. Moreover, because the PHQ-9, GAD-7, PCL-5, and PHQ-15 are self-report screening instruments rather than clinical diagnostic assessments, the clusters should not be interpreted as diagnostic categories.

Previous research has documented the co-occurrence and syndrome overlap of somatic and psychological symptoms in both clinical, primary care and population-based samples (3, 13, 18, 63–65). The present study adds to this literature by examining symptom patterns among young adults recruited through population-based sampling frameworks across geographically and contextually different settings, including populations affected by forced migration and conflict. Convergent findings have recently been reported in adolescents and young adults, where somatic and cognitive dimensions of psychological distress were strongly correlated and k-means clustering similarly identified high-, moderate-, and low-distress profiles (39). Similar severity-graded clustering has been reported in multidimensional symptom studies, including chronic pain and functional somatic disorders, where clusters were distinguished by overall symptom severity (66, 67). Additional work, examining youth anxiety and perceived stress characterizing heterogeneous symptom patterns and interrelations, further illustrates the value of multidimensional approaches to youth distress (14).

However, the present analyses cannot determine whether the observed correlations and symptom severity-based clusters reflect a broader dimension of general psychological distress, overlap between the measured symptom constructs, shared etiological or psychological processes, or a combination of these factors.

The observed pattern is therefore compatible with, but does not directly test, dimensional and transdiagnostic conceptualizations of mental health. Transdiagnostic approaches aim to characterize clinically relevant phenomena that extend across conventional diagnostic boundaries rather than being restricted to individual disorders (26). For example, the HiTOP framework organizes psychopathological symptoms according to their empirical patterns of covariation and represents psychopathology hierarchically along continuous dimensions (31–33, 37, 68). HiTOP is primarily a descriptive framework for organizing the phenotypic structure of psychopathology (25, 68).

Several processes may contribute to the co-occurrence of psychological and somatic symptoms. Previous studies suggest that the development of psychological and somatic symptoms are linked with emotion regulation difficulties, perceived stress, and stress reactivity (14, 69–73). Somatic complaints are prevalent in youth with emotional difficulties and among those diagnosed with psychiatric disorders, such as depression, trauma-related, and anxiety disorders (13, 40, 41, 74–76). Longitudinal evidence suggests that the relationship between somatic symptoms and psychological distress may be bidirectional, with somatic symptoms predicting subsequent psychological symptoms and psychological symptoms predicting subsequent somatic symptoms (77–80).

Contemporary aetiological models of somatic symptoms furthermore emphasize the interplay between physical and psychological processes and their complex interactions (81). These observations can also be considered in relation to emerging diagnostic models such as Bodily Distress Disorder (BDD) and Bodily Distress Syndrome (BDS) (19, 82–86). However, these diagnoses were not assessed in the present study and therefore the observed symptom patterns could not be interpreted as evidence for BDD or BDS.

As a secondary descriptive analysis, reported preexisting health conditions varied across the study samples, with Swiss young adults and Ukrainian refugees living in Switzerland reporting preexisting mental health conditions more frequently than Ukrainians living in Ukraine. As no inferential or adjusted between-group analyses were performed, the observed variations between study groups should be interpreted descriptively and may reflect differences in demographic and geographical-related characteristics rather than statistically established group differences. These differences may reflect structural barriers, greater stigma, and limited access to mental health care in Ukraine, even prior to the escalation of the war, potentially leading to underdiagnosis of mental health conditions and delayed help-seeking (87, 88). Studies in Switzerland showed an overall high mental health burden among Swiss adolescents, particularly in relation to the impact of the COVID-19 pandemic, as well as the influence of social media use (10, 89–91). Additionally, a vague conceptualization of chronic mental health conditions may have influenced responses among young adults (89). The increased vulnerability of refugee populations as reported in several studies (92–94), linked to war exposure and pre-migration as well as post-migration stressors (such as separation anxiety, resettlement challenges, and socioeconomic factors), may contribute to the reported preexisting mental health conditions among Ukrainian refugees in Switzerland.

From a clinical and public health perspective, our findings suggest that the assessment of young adults may benefit from considering the overall severity and co-occurrence of symptoms across psychological and somatic domains alongside disorder-specific screening. Severity-based symptom patterns may also help inform approaches to early identification and prevention planning that match the intensity of assessment and support to overall symptom burden and clinical need. More broadly, the findings support further evaluation of approaches that consider psychological and somatic symptoms of young adults jointly rather than treating these domains as entirely separate. Overall, these findings highlight the urgent need for population-level prevention strategies and public health policies aimed at reducing long-term mental health consequences in young adults.

Further research should clarify whether additional disorder-specific symptom configurations can be identified. Longitudinal studies are also needed to examine the stability of these symptom profiles over time and to determine whether individuals move between severity levels.

### Strengths and limitations

A strength of this study is the inclusion of large and diverse samples of young adults based on population registries. The use of established and widely applied screening instruments (PHQ-9, GAD-7, PCL-5, PHQ-15) enhances comparability with prior research and clinical practice. Furthermore, the study focuses specifically on young adulthood, a developmental period characterized by increased vulnerability to psychological and somatic symptom burden.

While this study provides valuable insights, there are several limitations. First, the cross-sectional analysis precludes causal inference regarding temporal relationships between somatic and psychological symptoms.

Second, between-group differences were examined descriptively, and no inferential statistical tests or adjusted comparative analyses were performed. The study samples differed in demographic, recruitment, migration-related, and socioeconomic characteristics. Observed variations across samples should therefore be interpreted as descriptive characteristics of the respective study samples rather than as statistically established or causal group differences.

Third, geographical region and migration status provide information on the broader conflict-related context but do not represent measures of individual war-related exposure. Participants living within the same geographical region may have experienced substantially different levels and types of war-related events. Accordingly, the present findings cannot establish associations between individual war-related exposure and psychological or somatic symptom burden.

Fourth, reliance on self-reported screening measures may introduce reporting bias and quantify symptom severity and therefore cannot replace clinical assessment. In addition, direct and conceptual overlap among symptoms assessed by the instruments may have increased correlations and contributed to the emergence of clusters differentiated primarily by overall severity. Furthermore, the PCL-5 was administered without a separate assessment of DSM-5 Criterion A. PCL-5 scores should therefore be interpreted as self-reported PTSD-related symptom severity rather than as evidence of clinically diagnosed PTSD or PTSD prevalence.

Fifth, differing recruitment strategies and contextual factors (e.g., displacement status, war exposure, potential digital divide) may influence reporting and limit comparability across samples.

Sixth, the cluster analysis was restricted to participants with complete and valid scores. This complete-case approach may have reduced statistical precision and introduced selection bias if score completeness was associated with participant characteristics or symptom severity. Also, applying k-means to numerous positively correlated symptom variables may favor solutions differentiated primarily by overall response magnitude.

Finally, recruitment through population-based sampling frameworks does not imply that the final analytical samples were representative of their respective underlying populations. Participation differed across study samples, no sampling or nonresponse weights were applied, and women were overrepresented, particularly in the Ukrainian sample. In addition, an exact response rate could not be determined for MAP-U. These factors may have introduced selection bias and have limited the generalizability. Accordingly, the findings should be interpreted as symptom patterns and associations observed within the study samples rather than as population-representative estimates.

## Conclusion

This study provides population-based evidence that somatic and psychological symptoms in young adults are highly correlated and that, across three geographically and contextually distinct populations, separate cluster analyses identified broadly similar patterns differentiated primarily by overall symptom burden, ranging from low to moderate and high severity. These findings are consistent with a dimensional interpretation of multi-symptom distress and highlight the need for considering overall symptom burden and the co-occurrence of psychological and somatic symptoms for diagnostic and prevention strategies to better capture the complexity of multisystem distress during this critical developmental period. Furthermore, our results underscore the importance of integrating somatic and psychological symptoms in prevention and early detection, by considering these domains jointly rather than as separate constructs. Further longitudinal and methodologically complementary research is needed to determine whether additional domain-specific symptom configurations emerge and to examine how symptom patterns develop and change over time.

## Data Availability

The data analyzed in this study is subject to the following licenses/restrictions: The datasets used and/or analyzed during the current study are available on reasonable request. Requests to access these datasets should be directed to marco.kaufmann@uzh.ch.
