# Supplementary material for: Psychological and somatic distress profiles in young adults in and out of conflict settings: a cluster-based analysis of three population-based studies in Ukraine and Switzerland
Source: Front Public Health. 2026 Sep 24;14:1922204. doi: 10.3389/fpubh.2026.1922204 (PMC13645757; doi:10.3389/fpubh.2026.1922204)
Supplement: Supplementary file 1 [file Data_Sheet_1.docx]

**Supplementary Material**

[Geographical overview of Ukrainian oblasts	2](#_Toc237789427)

[Sociodemographic characteristics and existing chronic health conditions of participants excluded from the analysis	3](#_Toc237789428)

[Number of missing values per screening instrument of the participants excluded from the analysis	4](#_Toc237789429)

[Direct and indirect war-related experiences reported by Ukrainian subgroups	4](#_Toc237789430)

[Cross-tabulation of somatic symptoms (PHQ-15) and symptoms related to depression, anxiety and post-traumatic stress disorder (PTSD)	5](#_Toc237789431)

[Frequency of the items of the PHQ-15	11](#_Toc237789432)

[Symptom burden (PHQ-15, PHQ-9, GAD-7 and PCL-5 sum scores) by study cohorts and oblasts (MAP-U)	14](#_Toc237789433)

[Descriptive results of PHQ-15 items stratified for symptom burden of PHQ-9, GAD-7 and PCL-5	15](#_Toc237789434)

Geographical overview of Ukrainian oblasts

To better reflect the different levels of war related stress exposure within Ukraine (MAP-U), we focused on three regional clusters formed from 10 selected oblasts across different parts of the country, each experiencing varying degrees of impact from the war (as described earlier) (1,2). During the time of recruitment, the North-West cluster (NW) (Rivne, Lviv, and Chernivtsi oblasts) was located far from the war zone and faced relatively low security risks. The Central cluster (CE) (Zhytomyr, Kyiv, and Dnipropetrovsk oblasts) was closer to the border with Russia and experienced moderate security risks. The South-East cluster (SE) (Sumy, Kharkiv, Mykolaiv, and Kherson oblasts) was situated near the war zone and faced high security risks due to its close proximity to the Russian border. **Figure S1** maps a geographical overview about the 10 selected oblasts grouped in three clusters (NW, CE and SE) based on their geographical distance to the war border and level of direct involvement in ongoing war activities.

**Figure S1.** Ukrainian oblasts included in the study and their clustering.

Sociodemographic characteristics and existing chronic health conditions of participants excluded from the analysis

Sociodemographic characteristics and existing chronic health conditions of young adults among the different subgroups are detailed in **Table S1**.

**Table S1**. Demographic characteristics and health conditions of MAP-Z (ZH), MAP-Z (UA), MAP-U age grouped 18-24 years old excluded from the analysis due to missing values

|  | **MAP-Z (ZH)**  (n=15) | **MAP-Z (UA)**  (n=11) | **MAP-U**  (n=213) | | |
| --- | --- | --- | --- | --- | --- |
|  |  |  | North-West  (n=63) | Central  (n=55) | South-East  (n=95) |
| **Sex** |  |  |  |  |  |
| Female | 10 (66.7%) | 8 (72.7%) | 53 (84.1%) | 46 (836%) | 72 (75.8%) |
| Male | 4 (26.7%) | 3 (27.3%) | 10 (15.9%) | 9 (16.4%) | 23 (24.2%) |
| Other | 1 (6.7%) | 0 (0.0%) | 0 (0.0%) | 0 (0.0%) | 0 (0%) |
| **Highest Educational Attainment** |  |  |  |  |  |
| Less than Secondary Education | 0 (0%) | 0 (0.0%) | 0 (0%) | 1 (1.8%) | 0 (0.0%) |
| Completed Secondary Education | 2 (13.3%) | 3 (27.3%) | 9 (14.3%) | 30 (54.5%) | 38 (40.0%) |
| Professional Education | 7 (46.7%) | 1 (9.1%) | 9 (14.3%) | 11 (20.0%) | 14 (14.7%) |
| Baccalaureate or higher professional education^1^ | 2 (13.3%) | NA | NA | NA | NA |
| University Degree | 4 (26.7%) | 7 (63.6%) | 44 (69.8%) | 12 (21.8%) | 43 (45.3%) |
| Missing | 0 (0%) | 0 (0%) | 1 (1.6%) | 1 (1.8%) | 0 (0%) |
| **Existing chronic physical conditions** |  |  |  |  |  |
| Yes^2^ | 1 (6.7%) | 4 (36.4%) | 20 (31.7%) | 17 (30.9%) | 25 (26.3%) |
| No | 14 (93.3%) | 7 (63.6%) | 42 (66.7%) | 37 (67.3%) | 69 (72.6%) |
| Missing | 0 (0%) | 0 (0%) | 1 (1.6%) | 1(1.8%) | 1 (1.1%) |
| **Existing chronic mental health conditions** |  |  |  |  |  |
| Yes | 1 (6.7%) | 2 (18.2%) | 1 (1.6%) | 2 (3.6%) | 4 (4.2%) |
| No | 14 (93.3%) | 9 (81.8%) | 61 (96.8%) | 52 (94.5%) | 90 (94.7%) |
| Missing | 0 (0%) | 0 (0%) | 1 (1.6%) | 1(1.8%) | 1 (1.1%) |

Number of missing values per screening instrument of the participants excluded from the analysis

**Table S2**. Overview of missing values of the screening instruments among participants excluded from the analysis.

| **Outcome** | **MAP-Z (ZH)** | **MAP-Z (UA)** | **MAP-U (NW)** | **MAP-U (CE)** | **MAP-U (SE)** |
| --- | --- | --- | --- | --- | --- |
| PHQ-15 | 8 | 1 | 17 | 19 | 24 |
| PHQ-9 | 4 | 1 | 5 | 4 | 7 |
| GAD-7 | 4 | 2 | 5 | 3 | 11 |
| PCL-5 | 8 | 1 | 11 | 16 | 24 |

Direct and indirect war-related experiences reported by Ukrainian subgroups

In **Table S3** we analyzed the distinct experiences reported by Ukrainian participants from different regions, providing background information on their living conditions and assumed war-related stress exposures. We summarized war-related experiences reported that participants have been directly involved as well indirectly through their relatives.

**Table S3.** Direct and indirect war-related experiences reported by MAP-Z (UA) and MAP-U (NW, CE and SE) participants, given as a number (percentage).

|  | **MAP-Z (UA)**  (n=91) | **MAP-U**  (n=963) | | |
| --- | --- | --- | --- | --- |
|  |  | North-West  (n=211) | Central  (n=230) | South-East  (n=522) |
| Displacement experience^1^ | 90 (98.9%)^2^ | 70 (33.2%) | 85 (37.0%) | 291 (55.7%) |
| Residence damaged | 14 (15.4%) | 8 (3.8%) | 18 (7.8%) | 106 (20.3%) |
| Relatives mobilized | 12 (13.2%) | 63 (29.9%) | 73 (31.7%) | 120 (23.0%) |
| Injuries or trauma from  hostilities^1^ | 8 (8.8%) | 16 (7.6%) | 37 (16.1%) | 67 (12.8%) |
| Gender-based violence^1^ | 3 (3.3%) | 6 (2.8%) | 6 (2.6%) | 17 (3.3%) |
| Death of relative | 14 (15.4%) | 34 (16.1%) | 24 (10.4%) | 67 (12.8%) |
| Separation from family members | 86 (94.5%) | 63 (29.9%) | 67 (29.1%) | 220 (42.1%) |

**Note.** ^1^Direct and indirect experiences have been summarized. ^2^Deviation from 100% may be explained by participants already living in Switzerland under different legal status or individual misunderstanding. Here presented as reported by participants for transparency. MAP-Z (UA): Mental Health Assessment of the Ukrainian Population in Zurich; MAP-U: Mental Health Assessment of the Population in Ukraine.

Cross-tabulation of somatic symptoms (PHQ-15) and symptoms related to depression, anxiety and post-traumatic stress disorder (PTSD)

We performed cross-tabulation analysis between psychological and somatic symptom severity across all young adult subgroups. PHQ-15 scores range from 0 to 30, with an established cut-point of ≥10 reflecting moderate to severe somatic symptom severity (3). This cut-off was identified as optimal to predict the diagnosis of a somatoform disorder in the primary care setting, resulting in a sensitivity of 0.80 and specificity of 0.59 (4,5). The moderate specificity reflects its design as a broad screening instrument for common somatic symptoms rather than a diagnostic tool. By capturing a wide range of physical complaints that frequently occur in outpatient and community settings, the PHQ-15 prioritizes sensitivity, which is appropriate for population-based research (4,5). PHQ-9 scores range from 0–27, with a cut-point of ≥10 indicating moderate to severe depression-related symptom severity (sensitivity 0.88, specificity 0.88) (6,7). For anxiety-related symptoms, a GAD-7 cut-point of ≥10 was applied (sensitivity 0.89, specificity 0.82) (8). PTSD-related symptom severity was dichotomized using a PCL-5 cut-point of ≥31 (sensitivity 0.94, specificity 0.94) (9). These cut-points were used to categorize symptom levels and do not represent clinical diagnoses.

The results indicate a high co-occurrence of moderate to severe somatic symptoms (defined as PHQ-15≥10) with moderate to severe depression-related, anxiety-related, and PTSD-related symptoms among all study arms (MAP-Z (ZH), MAP-Z (UA), MAP-U (NW), MAP-U (CE) and MAP-U (SE)) (**Tables S4-S8**).

**Table S4.** Cross-tabulation of moderate to severe somatic symptoms and elevated depression-, anxiety- and PTSD-related symptoms among MAP-Z (ZH).

|  | PCL-5 | |
| --- | --- | --- |
|  | **0** | **1** |
|  | N = 108^1^ | N = 19^1^ |
| GAD-7 |  |  |
| 0 | 92 (85%) | 6 (32%) |
| 1 | 16 (15%) | 13 (68%) |
| PHQ-9 |  |  |
| 0 | 76 (70%) | 3 (16%) |
| 1 | 32 (30%) | 16 (84%) |
| ^1^ n (%) |  |  |

|  | GAD-7 | |
| --- | --- | --- |
|  | **0** | **1** |
|  | N = 98^1^ | N = 29^1^ |
| PHQ-9 |  |  |
| 0 | 72 (73%) | 7 (24%) |
| 1 | 26 (27%) | 22 (76%) |
| ^1^ n (%) |  |  |

|  | PHQ-15 | |
| --- | --- | --- |
|  | **0** | **1** |
|  | N = 90^1^ | N = 37^1^ |
| PCL-5 |  |  |
| 0 | 81 (90%) | 27 (73%) |
| 1 | 9 (10%) | 10 (27%) |
| GAD-7 |  |  |
| 0 | 80 (89%) | 18 (49%) |
| 1 | 10 (11%) | 19 (51%) |
| PHQ-9 |  |  |
| 0 | 65 (72%) | 14 (38%) |
| 1 | 25 (28%) | 23 (62%) |
| ^1^ n (%) |  |  |

**Table S5.** Cross-tabulation of moderate to severe somatic symptoms and elevated depression-, anxiety-, and PTSD-related symptoms among MAP-Z (UA).

|  | PCL-5 | |
| --- | --- | --- |
|  | **0** | **1** |
|  | N = 56^1^ | N = 35^1^ |
| GAD-7 |  |  |
| 0 | 50 (89%) | 14 (40%) |
| 1 | 6 (11%) | 21 (60%) |
| PHQ-9 |  |  |
| 0 | 38 (68%) | 3 (8.6%) |
| 1 | 18 (32%) | 32 (91%) |
| ^1^ n (%) |  |  |

|  | GAD-7 | |
| --- | --- | --- |
|  | **0** | **1** |
|  | N = 64^1^ | N = 27^1^ |
| PHQ-9 |  |  |
| 0 | 37 (58%) | 4 (15%) |
| 1 | 27 (42%) | 23 (85%) |
| ^1^ n (%) |  |  |

|  | PHQ-15 | |
| --- | --- | --- |
|  | **0** | **1** |
|  | N = 45^1^ | N = 46^1^ |
| PCL-5 |  |  |
| 0 | 39 (87%) | 17 (37%) |
| 1 | 6 (13%) | 29 (63%) |
| GAD-7 |  |  |
| 0 | 40 (89%) | 24 (52%) |
| 1 | 5 (11%) | 22 (48%) |
| PHQ-9 |  |  |
| 0 | 33 (73%) | 8 (17%) |
| 1 | 12 (27%) | 38 (83%) |
| ^1^ n (%) |  |  |

**Table S6.** Cross-tabulation of moderate to severe somatic symptoms and elevated depression-, anxiety-, and PTSD-related symptoms among MAP-U (NW).

|  | PCL-5 | |
| --- | --- | --- |
|  | **0** | **1** |
|  | N = 136^1^ | N = 75^1^ |
| GAD-7 |  |  |
| 0 | 126 (93%) | 33 (44%) |
| 1 | 10 (7.4%) | 42 (56%) |
| PHQ-9 |  |  |
| 0 | 112 (82%) | 15 (20%) |
| 1 | 24 (18%) | 60 (80%) |
| ^1^ n (%) |  |  |

|  | GAD-7 | |
| --- | --- | --- |
|  | **0** | **1** |
|  | N = 159^1^ | N = 52^1^ |
| PHQ-9 |  |  |
| 0 | 118 (74%) | 9 (17%) |
| 1 | 41 (26%) | 43 (83%) |
| ^1^ n (%) |  |  |

|  | PHQ-15 | |
| --- | --- | --- |
|  | **0** | **1** |
|  | N = 128^1^ | N = 83^1^ |
| PCL-5 |  |  |
| 0 | 101 (79%) | 35 (42%) |
| 1 | 27 (21%) | 48 (58%) |
| GAD-7 |  |  |
| 0 | 110 (86%) | 49 (59%) |
| 1 | 18 (14%) | 34 (41%) |
| PHQ-9 |  |  |
| 0 | 101 (79%) | 26 (31%) |
| 1 | 27 (21%) | 57 (69%) |
| ^1^ n (%) |  |  |

**Table S7.** Cross-tabulation of moderate to severe somatic symptoms and elevated depression-, anxiety-, and PTSD-related symptoms MAP-U (CE).

|  | PCL-5 | |
| --- | --- | --- |
|  | **0** | **1** |
|  | N = 145^1^ | N = 85^1^ |
| GAD-7 |  |  |
| 0 | 125 (86%) | 26 (31%) |
| 1 | 20 (14%) | 59 (69%) |
| PHQ-9 |  |  |
| 0 | 112 (77%) | 15 (18%) |
| 1 | 33 (23%) | 70 (82%) |
| ^1^ n (%) |  |  |

|  | GAD-7 | |
| --- | --- | --- |
|  | **0** | **1** |
|  | N = 151^1^ | N = 79^1^ |
| PHQ-9 |  |  |
| 0 | 112 (74%) | 15 (19%) |
| 1 | 39 (26%) | 64 (81%) |
| ^1^ n (%) |  |  |

|  | PHQ-15 | |
| --- | --- | --- |
|  | **0** | **1** |
|  | N = 116^1^ | N = 114^1^ |
| PCL-5 |  |  |
| 0 | 98 (84%) | 47 (41%) |
| 1 | 18 (16%) | 67 (59%) |
| GAD-7 |  |  |
| 0 | 100 (86%) | 51 (45%) |
| 1 | 16 (14%) | 63 (55%) |
| PHQ-9 |  |  |
| 0 | 92 (79%) | 35 (31%) |
| 1 | 24 (21%) | 79 (69%) |
| ^1^ n (%) |  |  |

**Table S8.** Cross-tabulation of moderate to severe somatic symptoms and elevated depression-, anxiety-, and PTSD-related symptoms among MAP-U (SE).

|  | PCL-5 | |
| --- | --- | --- |
|  | **0** | **1** |
|  | N = 327^1^ | N = 195^1^ |
| GAD-7 |  |  |
| 0 | 282 (86%) | 57 (29%) |
| 1 | 45 (14%) | 138 (71%) |
| PHQ-9 |  |  |
| 0 | 242 (74%) | 37 (19%) |
| 1 | 85 (26%) | 158 (81%) |
| ^1^ n (%) |  |  |

|  | GAD-7 | |
| --- | --- | --- |
|  | **0** | **1** |
|  | N = 339^1^ | N = 183^1^ |
| PHQ-9 |  |  |
| 0 | 249 (73%) | 30 (16%) |
| 1 | 90 (27%) | 153 (84%) |
| ^1^ n (%) |  |  |

|  | PHQ-15 | |
| --- | --- | --- |
|  | **0** | **1** |
|  | N = 288^1^ | N = 234^1^ |
| PCL-5 |  |  |
| 0 | 236 (82%) | 91 (39%) |
| 1 | 52 (18%) | 143 (61%) |
| Unknown | 3 | 1 |
| GAD-7 |  |  |
| 0 | 236 (82%) | 103 (44%) |
| 1 | 52 (18%) | 131 (56%) |
| PHQ-9 |  |  |
| 0 | 213 (74%) | 66 (28%) |
| 1 | 75 (26%) | 168 (72%) |
| ^1^ n (%) |  |  |

Frequency of the items of the PHQ-15

**Figures S2-S4** show the frequency of the single items of the PHQ-15 items reported in the Zurich (MAP-Z (ZH), MAP-Z (UA)) and Ukrainian (MAP-U (NW), MAP-U (CE) and MAP-U (SE)) samples. The most frequently reported somatic complaints among all subgroups were fatigue, sleep difficulties, back pain and headaches. For the Ukrainian subgroups in Switzerland as well as in Ukraine (**Figures S3-S6**) the symptoms heart racing and shortness of breath were more frequently reported. A stacked barplot of PHQ-15 item responses revealed considerable heterogeneity in symptom expression.

**Figure S2**. Frequency of the single PHQ-15 items in MAP-Z (ZH).

**Figure S3**. Frequency of the single PHQ-15 items in MAP-Z (UA).

**Figure S4**. Frequency of the single PHQ-15 items in MAP-U (NW).

**Figure S5**. Frequency of the single PHQ-15 items in MAP-U (CE).

**Figure S6**. Frequency of the single PHQ-15 items in MAP-U (SE).

Symptom burden (PHQ-15, PHQ-9, GAD-7 and PCL-5 sum scores) by study cohorts and oblasts (MAP-U)

**Table S9** illustrates the corresponding descriptive statistics. For each group, it reports the median, the first and third quartiles (Q1 and Q3), and the minimum and maximum non-outlier values.

**Table S9**. PHQ-15, PHQ-9, GAD-7 and PCL-5 sum scores by study cohorts.

| Outcome | Study arm | n | min. | Q1 | median | Q3 | max. |
| --- | --- | --- | --- | --- | --- | --- | --- |
| PHQ-15 | MAP-Z (ZH) | 127 | 0 | 4 | 6 | 10 | 22 |
| PHQ-15 | MAP-Z (UA) | 91 | 2 | 5 | 10 | 13 | 30 |
| PHQ-15 | MAP-U (NW) | 211 | 0 | 5 | 8 | 12 | 23 |
| PHQ-15 | MAP-U (CE) | 230 | 0 | 6 | 9 | 13 | 22 |
| PHQ-15 | MAP-U (SE) | 522 | 0 | 5 | 9 | 13 | 27 |
| PHQ-9 | MAP-Z (ZH) | 127 | 0 | 4 | 8 | 11 | 22 |
| PHQ-9 | MAP-Z (UA) | 91 | 0 | 6 | 10 | 14 | 25 |
| PHQ-9 | MAP-U (NW) | 211 | 0 | 4 | 8 | 12 | 27 |
| PHQ-9 | MAP-U (CE) | 230 | 0 | 5 | 8 | 13.8 | 27 |
| PHQ-9 | MAP-U (SE) | 522 | 0 | 5 | 9 | 14 | 27 |
| GAD-7 | MAP-Z (ZH) | 127 | 0 | 2.5 | 5 | 9 | 19 |
| GAD-7 | MAP-Z (UA) | 91 | 0 | 4 | 7 | 11.5 | 21 |
| GAD-7 | MAP-U (NW) | 211 | 0 | 3 | 6 | 9 | 21 |
| GAD-7 | MAP-U (CE) | 230 | 0 | 4 | 6 | 11 | 21 |
| GAD-7 | MAP-U (SE) | 522 | 0 | 4 | 7 | 12 | 21 |
| PCL-5 | MAP-Z (ZH) | 127 | 0 | 4 | 11 | 24 | 65 |
| PCL-5 | MAP-Z (UA) | 91 | 0 | 15.5 | 26 | 39 | 60 |
| PCL-5 | MAP-U (NW) | 211 | 0 | 10 | 22 | 36 | 67 |
| PCL-5 | MAP-U (CE) | 230 | 0 | 11 | 23 | 37 | 80 |
| PCL-5 | MAP-U (SE) | 522 | 0 | 14 | 24 | 38.8 | 80 |

Descriptive results of PHQ-15 items stratified for symptom burden of PHQ-9, GAD-7 and PCL-5

**Figures S7-S11** present the distribution of symptoms related to depression (PHQ-9), anxiety (GAD-7), and PTSD (PCL-5) scores by severity of each somatic symptom (PHQ-15 items) across the three PHQ-15 response categories (“not bothered at all”, “bothered a little”, “bothered a lot”) for each of the 15 somatic symptoms among all subgroups. Across all subgroups and the three mental health outcomes, higher somatic symptom severity was associated with higher psychological symptom scores. Participants who reported being “bothered a lot” by a given somatic symptom generally exhibited the highest median scores for depression-, anxiety-, and PTSD-related symptoms. Overall, these analyses reveal a symptom-specific graded association between the severity of somatic complaints and the burden of psychological symptoms.

**Figure S7.** Boxplots of PHQ-9, GAD-7 and PCL-5, stratified by somatic symptom severity for MAP-Z (ZH).

**Figure S8**. Boxplots of PHQ-9, GAD-7 and PCL-5, stratified by somatic symptom severity for MAP-Z (UA).

**Figure S9**. Boxplots of PHQ-9, GAD-7 and PCL-5, stratified by somatic symptom severity for MAP-U (NW).

**Figure S10**. Boxplots of PHQ-9, GAD-7 and PCL-5, stratified by somatic symptom severity for MAP-U (CE).

**Figure S11**. Boxplots of PHQ-9, GAD-7 and PCL-5, stratified by somatic symptom severity for MAP-U (SE).

**References**

1.	Yasenok V, Baumer AM, Petrashenko V, Kaufmann M, Frei A, Rüegger S, Ballouz T, Loboda A, Smiianov V, Seifritz E, et al. Mental health burden of persons living in Ukraine and Ukrainians displaced to Switzerland: the mental health assessment of the Ukrainian population (MAP) studies. *BMJ Glob Health* (2025) 10:e019557. doi: 10.1136/bmjgh-2025-019557

2.	Yasenok V, Neumann E, Raineri A, Kopp J, Rüegger S, Ballouz T, Kaufmann M, Loboda A, Smiianov V, Baumer AM, et al. Mental Health Assessment of the Population: Study Protocol of the MAP Research Program in Ukraine (MAP-U) and in Zurich (MAP-Z). *Int J Public Health* (2025) 69: doi: 10.3389/ijph.2024.1607271

3.	Kroenke K, Spitzer RL, Williams JBW. The PHQ-15: Validity of a New Measure for Evaluating the Severity of Somatic Symptoms. *Psychosom Med* (2002) 64:258–266. doi: 10.1097/00006842-200203000-00008

4.	Körber S, Frieser D, Steinbrecher N, Hiller W. Classification characteristics of the Patient Health Questionnaire-15 for screening somatoform disorders in a primary care setting. *J Psychosom Res* (2011) 71:142–147. doi: 10.1016/j.jpsychores.2011.01.006

5.	Kocalevent R-D, Hinz A, Brähler E. Standardization of a screening instrument (PHQ-15) for somatization syndromes in the general population. *BMC Psychiatry* (2013) 13:91. doi: 10.1186/1471-244X-13-91

6.	Kroenke K, Spitzer RL, Williams JBW. The PHQ-9. *J Gen Intern Med* (2001) 16:606–613. doi: 10.1046/j.1525-1497.2001.016009606.x

7.	Kroenke K, Spitzer RL, Williams JBW, Löwe B. The Patient Health Questionnaire Somatic, Anxiety, and Depressive Symptom Scales: a systematic review. *Gen Hosp Psychiatry* (2010) 32:345–359. doi: 10.1016/j.genhosppsych.2010.03.006

8.	Spitzer RL, Kroenke K, Williams JBW, Löwe B. A Brief Measure for Assessing Generalized Anxiety Disorder. *Arch Intern Med* (2006) 166:1092. doi: 10.1001/archinte.166.10.1092

9.	Geier TJ, Hunt JC, Nelson LD, Brasel KJ, deRoon-Cassini TA. Detecting PTSD in a traumatically injured population: The diagnostic utility of the PTSD Checklist for DSM-5. *Depress Anxiety* (2019) 36:170–178. doi: 10.1002/da.22873
